# Analytical Performance and Inter-Method Agreement of a Laboratory-Developed CMV qPCR Assay in Clinical Plasma Samples

**DOI:** 10.3390/microorganisms14051127

**Published:** 2026-05-15

**Authors:** Murat Aral, Ayfer Bakır, Cemal Çiçek, Elif Tuğçe Güner, Didem Özkan, Muhammed Furkan Kürkçü, Gülşah Ceylan Yağız, Mehmet Morkoç, Ferit Kulalı, Ahmet Kürşad Güneş

**Affiliations:** 1 Department of Medical Microbiology, Ankara Etlik City Hospital, Ankara 06170, Türkiye; dr.ayfer.bakir@gmail.com (A.B.); drcemal06@gmail.com (C.Ç.); eliftugce06md@gmail.com (E.T.G.); didem.yigitt@gmail.com (D.Ö.); furkankurkcu@gmail.com (M.F.K.); ceylan218@outlook.com (G.C.Y.); mehmet.morkoc93@gmail.com (M.M.); 2 Department of Neonatology, Ankara Etlik City Hospital, Ankara 06170, Türkiye; fkulali@hotmail.com; 3 Department of Hematology, Ankara Etlik City Hospital, Ankara 06170, Türkiye; ahmetkgunes@gmail.com

**Keywords:** cytomegalovirus, real-time quantitative PCR, laboratory-developed test, analytical performance, method comparison

## Abstract

Background: Cytomegalovirus (CMV) viral load monitoring forms the basis of preemptive treatment strategies in patients undergoing solid organ and hematopoietic stem cell transplantation. This study aimed to evaluate the analytical performance and inter-method agreement of a laboratory-developed CMV real-time PCR (qPCR) test compared to a commercial reference method using plasma samples. Methods: A total of 100 EDTA plasma samples were analyzed in parallel using a laboratory-developed CMV qPCR test and the reference method (Roche Cobas^®^ CMV). Analytical sensitivity was determined us-ing synthetic DNA cloned into the pUC57 plasmid backbone containing the US17 region of the CMV genome, and the limit of detection (LoD_95_) was calculated using probit regression analysis. The relationship between the quantitative results obtained from clinical samples was evaluated using the Spearman rank correlation coefficient, while inter-method clinical agreement was assessed using the Bland–Altman method. Results: The limit of detection (LoD_95_) of the laboratory-developed CMV qPCR test, as determined by probit regression analysis, was 63.8 copies/µL. A weak and statistically non-significant correlation was ob-served between the laboratory-developed CMV qPCR test and the reference method in Spearman rank correlation analysis of samples for which numerical quantitative results were available from both methods (ρ = 0.32; *p* = 0.22; *n* = 16). Bland–Altman analysis showed a mean difference of −0.48 log_10_ units, with the vast majority of measurements falling within the 95% limits of agreement. Conclusions: The assay demonstrated measurable analytical performance and inter-method agreement; however, its use for quantitative viral load monitoring, particularly at low CMV DNA levels, should be interpreted with caution.

## 1. Introduction

Cytomegalovirus (CMV) is a common opportunistic pathogen belonging to the Her-pesviridae family, causing significant morbidity and mortality, particularly in immuno-compromised individuals such as solid organ transplant and hematopoietic stem cell transplant recipients [1,2,3]. In this patient group, CMV infection can cause direct viral syndromes and tissue-invasive diseases (such as hepatitis, pneumonia, retinitis, and gastroenteritis), as well as indirect effects that negatively impact transplant outcomes [3,4,5]. These indirect effects include increased infection frequency, graft loss, acute and chronic rejection, cardiovascular events, and decreased patient and allograft survival [3,4,6]. Especially CMV pneumonia, with reported mortality rates exceeding 50%, presents a serious clinical picture [7].

Clinical management of CMV infection in this patient group is largely based on prophylaxis and preemptive treatment strategies relying on viral load monitoring [8,9,10]. The effectiveness of preemptive treatment approaches depends on quantitative real-time PCR (qPCR) methods that can reliably measure, especially at low viral load levels. Clinical studies have shown significant relationships between CMV viral load levels and disease progression, and that serial viral load measurements provide higher predictive power in clinical decision-making processes compared to a single measurement [8,11]. However, re-ported measurement differences between different CMV qPCR tests can arise depending on the choice of target gene region, primer-probe design, calibration strategies, and the analytical platforms used [12,13].

The international standard for CMV DNA defined by the World Health Organization (WHO) has made it possible to report viral load results in IU/mL [14]. However, it has been reported that the conversion between IU and copy number is method-specific, can vary de-pending on the target gene region and calibrator structure, and therefore absolute quantita-tive equivalence cannot always be achieved between methods [13,15].

This situation makes it difficult to compare between laboratories and standardize clinical thresholds. Therefore, before integrating laboratory-developed CMV qPCR tests into clinical use, their analytical sensitivity and clinical performance must be evaluated and compared to reference methods. The aim of this study is to evaluate the analytical and clinical performance of a laboratory-developed CMV quantitative qPCR test compared to a commercial reference test in clinical plasma samples.

## 2. Materials and Methods

### 2.1. Study Population and Clinical Samples

This study was conducted between 29 January 2026 and 20 February 2026 at the Medical Microbiology Laboratory of Ankara Etlik City Hospital, using a prospective, cross-sectional, and analytical design. The study population consists of plasma samples from patients with a preliminary or confirmed diagnosis of CMV infection who were requested for quantitative CMV DNA analysis and were sent to the laboratory from the pediatric health and diseases clinic, pediatric neonatal unit, pediatric bone marrow transplantation unit, pediatric infectious diseases unit, adult bone marrow transplantation unit, and adult hematology-oncology clinics. Only EDTA plasma samples taken during routine clinical practice were used in the study, and no additional intervention was applied to the participants. Informed consent was obtained from all participants or their legal representatives; patient identifying information was kept confidential and the data was anonymized. The analyzed plasma samples were obtained from patients undergoing CMV testing as part of routine clinical follow-up, including individuals from solid organ transplantation, hematopoietic stem cell transplantation, and hematology–oncology patient groups.

Blood samples were delivered to the laboratory under appropriate pre-analytical transport conditions. EDTA blood samples accepted into the laboratory were centrifuged at 1500× *g* for 10 min at room temperature to separate the plasma fraction. The obtained plasma samples were aliquoted to avoid contamination and freeze-thaw cycles and stored frozen at −80 °C until the time of analysis. All samples were stored in single-use aliquots throughout the study period.

All plasma samples were analyzed in parallel and simultaneously using the devel-oped CMV real-time qPCR test and a commercial reference method. Samples were anony-mized before analysis, and samples with extraction or internal control failures were ex-cluded from evaluation.

In this study, the sample size was determined according to a method comparison design for evaluating the analytical and comparative performance of a CMV real-time qPCR assay implemented in our laboratory using the Pharmaline CMV qPCR reagent system (Pharmaline Health Services Industrial and Commercial Inc., Istanbul, Türkiye). A total of 100 EDTA plasma samples obtained during routine clinical practice and meeting the inclusion criteria for the study were considered sufficient for the reliable evaluation of the agreement, correlation, and diagnostic performance parameters between the developed test and the commercial reference method.

Ethical Approval: The study was conducted in accordance with the Declaration of Helsinki and was approved by the Scientific Research Evaluation and Ethics Committee of Ankara Etlik City Hospital (Approval No: AEŞH-BADEK-2026-125; Date: 28 January 2026).

### 2.2. Inclusion and Exclusion Criteria

Samples with a sufficient volume of EDTA plasma between the specified dates and complete pre-analytical and analytical processes were included in the study. Clinical sam-ple types other than plasma, samples with insufficient volume or significant hemolysis, samples delivered to the laboratory without proper transport conditions, and samples with pre-analytical errors detected during centrifugation, aliquoting, or storage processes were excluded from the study. Additionally, samples that failed during nucleic acid extraction or did not undergo internal control amplification were excluded from analysis.

### 2.3. CMV DNA Analysis

Nucleic Acid Extraction: Total nucleic acid extraction from clinical samples was per-formed using a fully automated extraction system. Extraction procedures were carried out according to the manufacturer’s instructions using the Molecision Fully Automated Nucleic Acid Purification System (Molecision Biotech, Shenzhen, China) and the SNIBE Nucleic Acid Extraction Kit (SNIBE Diagnostics, Shenzhen, China). A volume of 200 µL of plasma was used for nucleic acid extraction and eluted in a final volume of 50 µL. Extraction efficiency and potential PCR inhibition were monitored by internal control amplification.

For analytical sensitivity studies, the limit of detection (LoD) was calculated based on the concentration of CMV DNA in the eluate and was therefore expressed as copies/µL. Considering that 200 µL of plasma was eluted in 50 µL, the LoD_95_ of 63.8 copies/µL corre-sponds to approximately 1.6 × 10^4^ copies/mL of plasma.

Real-Time PCR (qPCR) Design: Real-time qPCR amplifications were performed using the Bio-Rad CFX96 Touch Real-Time PCR Detection System (Bio-Rad Laboratories, Hercules, CA, USA). In PCR reactions, RapidXFire™ qPCR Master Mix (LGC Biosearch Technologies, San Diego, CA, USA; REF: 30050-100ML; LOT: 1606371) was used at a 2× final concentration. In real-time PCR analyses, the highly conserved US17 gene region of the CMV genome was targeted. The following sequences were used for the amplification of CMV DNA: forward primer (5′-TCTCTGTACCTCCCGCAAAA-3′), reverse primer (5′-AGACAAACTCATCGCT TGGA-3′), and probe (5′-FAM-TGACCTGGTTATCGTCACGCG-BHQ-3′).

US17 Region and In Silico Analysis: To assess the conservation of the US17 target region, an in silico alignment analysis was performed using available CMV genome sequences. The analysis demonstrated the absence of significant mismatches within the primer and probe binding regions, supporting the sequence compatibility and analytical specificity of the primer–probe set used in this study. Detailed alignment results are provided as Supplementary Material (Table S1).

To monitor the extraction and amplification processes, the human β-actin gene was included in the reactions as an internal control. The reaction conditions were optimized to final concentrations of 500 nM for CMV primers and 125 nM for the CMV probe; and 250 nM for β-actin primers and 62.5 nM for the β-actin probe. qPCR reactions were prepared in a total volume of 20 µL, with each reaction containing 10 µL of 2× qPCR Master Mix, 5 µL of primer/probe mix, and 5 µL of nucleic acid. Each work set includes a DNA-free negative control (NTC) and a negative control containing only the primer/probe mix (NK) for contamination monitoring. The thermal cycling conditions were applied in 44 cycles, consisting of an initial denaturation at 95 °C for 5 min, followed by denaturation at 95 °C for 10 s and binding/extension at 60 °C for 45 s. Fluorescence signal acquisition was performed at each binding/extension step of the cycle. The assay demonstrated a linear working range between 10^6^ and 10^2^ copies/µL, with Ct values decreasing in a log-linear manner across serial dilutions. Technical replicate variation was low across the tested concentration range, indicating acceptable intra-assay repeatability. Inter-assay reproducibility was supported by day-to-day Ct differences generally remaining ≤ 0.5 cycles across two experimental runs. The experimental design, execution, and reporting were conducted in accordance with the principles outlined in the Minimum Information for Publication of Quantitative Real-Time PCR Experiments (MIQE) guidelines, and the MIQE 2.0 checklist is provided as Supplementary Material (Supplementary Table S2) [16].

### 2.4. Reference CMV PCR Method

In the performance evaluation of the laboratory-developed CMV qPCR test, the commercial-ly validated and widely used cobas^®^ CMV test (Roche Diagnostics, Mannheim, Germany) was accepted as the reference method. The cobas^®^ CMV test is a fully automated real-time PCR test designed for the quantitative detection of CMV DNA, providing results in IU/mL traceable to the World Health Organization’s (WHO) 1st International CMV Standard. The tests were performed on the cobas^®^ 8800 system, following the manufacturer’s recommended protocol. The cobas^®^ CMV test targets the UL54 gene region and has high analytical sensitivity and accuracy.

### 2.5. Statistical Analysis

Statistical analyses were performed using IBM SPSS Statistics v26.0 (IBM Corp., Armonk, NY, USA) software to evaluate the analytical and clinical performance of the newly developed CMV qPCR kit. In the evaluation of analytical performance, a synthetic DNA construct cloned in-to the pUC57 (GenScript, Piscataway, NJ, USA) plasmid backbone, containing the targeted region of the CMV genome, was used. Analytical sensitivity was evaluated according to CLSI EP17-A2 guidelines; the relationship between the probability of detection and the target concentration was analyzed using a probit regression model [17].

The pUC57 plasmid, synthesized by GenScript (USA) and containing the target CMV sequence, was used as a reference material in calibration and analytical performance studies. The plasmid DNA concentration provided by the manufacturer was used to estimate the copy number of the construct, and serial dilutions were prepared to generate a calibration range between 10^6^ and 10^2^ copies/µL for evaluation of amplification efficiency and linearity. Additionally, Amplirun Cytomegalovirus DNA Control (Vircell Microbiologists, Granada, Spain; Ref: MBC016, Lot: 24MBC016001-C; 17,000 copies/µL) was used as a quantitative reference material. Intermediate concentrations prepared from this material (1700, 170, and 17 copies/µL) were included as calibration checkpoints to confirm the consistency of Ct values across the dynamic range of the assay. To assess the reproducibility of the calibration and to evaluate day-to-day variability, the experiments were repeated on two different days using three different primer–probe lots. Technical replicate variation was low, and day-to-day Ct differences were generally ≤0.5 cycles, indicating good repeatability and inter-run reproducibility of the assay.

Analytical sensitivity (limit of detection, LoD) experiments were performed using purified DNA reference materials diluted to defined concentrations and analyzed directly by qPCR. LoD studies were conducted in two sequential phases. In the initial screening phase, concentrations between 5 and 35 copies/µL were tested to identify the approximate concentration range in which the probability of detection approached the assay’s detection limit. In the second, confirmatory phase, concentrations between 40 and 100 copies/µL were analyzed in 24 replicate reactions across two consecutive experimental days to obtain robust estimates of detection probability. Probit regression analysis was used to model the relationship between CMV DNA concentration and the probability of detection and to estimate the concentration corresponding to a 95% probability of detection (LoD_95_). In the confirmatory experiments, a detection rate above 95% was observed at 50 copies/µL. Based on probit regression modelling, the LoD_95_ value was estimated to be 63.8 copies/µL. In addition, calibration experiments demonstrated that all replicates yielded positive amplification at 10^2^ copies/µL, whereas inconsistent amplification was observed at 10^1^ copies/µL, indicating that this concentration range approached the analytical detection limit of the assay.

The relationship between the quantitative results obtained with the reference test (cobas^®^ CMV) and the laboratory-developed CMV qPCR assay was evaluated using the Spearman rank correlation coefficient (ρ) due to the different reporting units of the results (cobas^®^ CMV: IU/mL; laboratory-developed assay: copies/µL), the non-normal distribution of the viral load data, and the analysis after applying a log_10_ transformation. The Spearman correlation coefficient and its corresponding *p*-values were calculated. Spearman correlation analysis was restricted to samples with numerical quantitative results reported by both methods. Although calibration materials traceable to the WHO International Standard for CMV were used, IU–copies conversion factors were not calculated because such factors are assay-dependent and not universally transferable. Nevertheless, individual quantitative results from both methods are provided side by side in Supplementary Table S5, allowing readers to directly inspect inter-method differences on a per-sample basis despite the unit discrepancy. The probit regression model used for LoD estimation was fitted using log10-transformed CMV DNA concentration as the predictor variable and binary detection outcomes (positive/negative) as the response variable. Model parameters were estimated using maximum likelihood estimation in SPSS.

Bland-Altman difference analysis was applied to assess whether the measurement differences between the two methods were within clinically acceptable limits. Within the scope of this analysis, the measurement difference between the two methods (new kit—ref-erence test) and the average of the measurements were calculated for each sample; the mean difference (bias) and 95% limits of agreement (LoA) were determined. In the Bland-Altman analysis, the bias value remaining within the limits of ±0.5 log_10_ IU/mL was considered clinically acceptable method agreement [18]. Bland–Altman agreement analysis additionally included paired detectable results after log_10_ transformation, including values below the limit of quantification.

## 3. Results

### 3.1. Analytical Sensitivity

**Figure 1:**
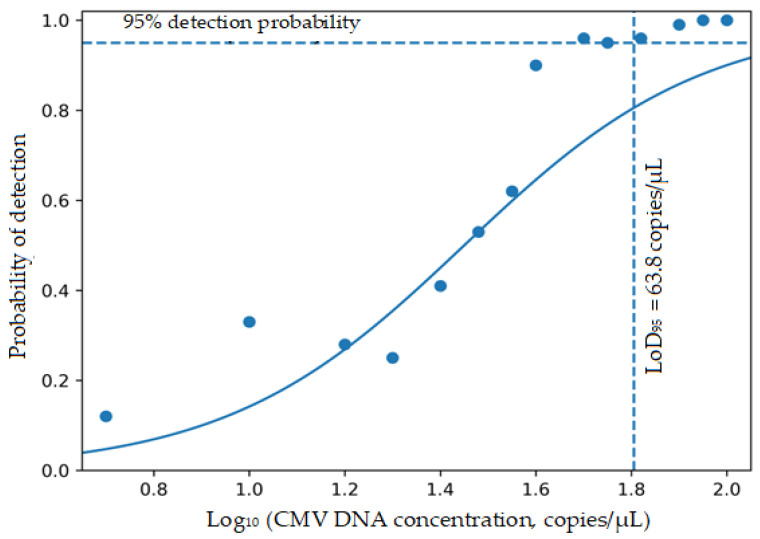
Probit dose–response curve for estimation of the limit of detection (LoD_95_) of the CMV qPCR assay.

The LoD95 for the CMV qPCR test, as determined by probit regression analysis, was 63.8 copies/µL, corresponding to a 95% probability of detection (Table 1, Figure 1). In the confirmatory LoD experiments, a detection rate of 95.83% (23/24 replicates) was observed at 50 copies/µL. This observation supported the LoD_95_ estimate of 63.8 copies/µL obtained from probit regression modelling.

The relationship between target concentration and detection probability was evaluated using a probit regression model, and all model parameters were found to be statistically significant (*p* < 0.001). Accordingly, the LoD_95_ corresponding to a 95% detection probability of the test was determined to be 63.8 copies/µL.

Detection probabilities (positive replicates/total replicates) obtained from 24 replicate reactions at each concentration level were plotted against log10-transformed CMV DNA concentrations (copies/µL). The solid curve represents the fitted probit regression model. The horizontal dashed line indicates a 95% detection probability, and the vertical dashed line represents the estimated LoD95 value (63.8 copies/µL).

In the agreement analysis between the two methods, the positive percent agreement (PPA), negative percent agreement (NPA), and overall percent agreement (OPA) values were calculated. The agreement results between the two assays are summarized in Table 2.

A total of 100 clinical samples were analyzed using the laboratory-developed CMV qPCR assay and the commercial Roche cobas^®^ CMV test. The Roche assay was used as the reference method. In the Roche test, samples reported as <34.5 IU/mL and detected below the limit of quantification, in accordance with the manufacturer’s instructions for use, were considered positive. According to this definition, 23 of the 37 samples classified as positive by the Roche assay were also detected as positive by the laboratory-developed assay, while 14 samples were negative by the laboratory-developed assay and were therefore classified as false negatives (FN). Among the 63 samples reported as negative by the Roche assay, 58 yielded concordant negative results with both methods, whereas 5 samples were classified as false positives (FP). False-negative results were predominantly observed in samples with low-level viral loads close to the assay’s detection limit.

Based on this comparison, the overall agreement between the two methods was 81.0% (95% CI: 72.2–87.5). The positive percent agreement (PPA) was 62.2% (95% CI: 46.1–75.9), while the negative percent agreement (NPA) was 92.1% (95% CI: 82.7–96.6). Detailed diagnostic performance metrics, including Cohen’s kappa statistics, are provided in Supplementary Tables S3 and S4.

Individual CMV viral load results (IU/mL and copies/mL) obtained by both methods are presented in Supplementary Table S5.

### 3.2. Quantitative Clinical Comparison

Quantitative results suitable for log_<_ transformation were obtained from both methods in 16 plasma samples and were included in the Spearman rank correlation analysis. Samples reported as <34.5 IU/mL or N/A were excluded from the correlation analysis.

In these 16 plasma samples, a weak and statistically non-significant correlation was observed between the laboratory-developed CMV qPCR test and the Roche cobas^®^ CMV test (ρ = 0.324; *p* = 0.221).

Bland–Altman agreement analysis was performed on a larger dataset (*n* = 21) by including paired detectable results after log10 transformation to assess the magnitude of differences between the two methods. The mean difference between the two methods was −0.48 log10 units, and the majority of measurements fell within the 95% limits of agreement.

The identifiers of samples included in the Spearman correlation (*n* = 16) and Bland–Altman agreement analyses (*n* = 21) are provided in Supplementary Table S6.

### 3.3. Inter-Method Agreement

Inter-method agreement between the laboratory-developed CMV qPCR assay and the reference method was further evaluated using Bland–Altman analysis (Table 3). The analysis was performed on paired quantitative results after log10 transformation to assess the magnitude of differences between the two methods.

## 4. Discussion

CMV viral load monitoring is one of the fundamental components of infection management in patients undergoing solid organ and hematopoietic stem cell transplantation, and plays a crucial role in determining the timing of preemptive treatment strategies [1,2,8,9,10,11]. However, the findings of this study suggest that the assay may have limited utility in detecting low-level viremia and in precise viral load monitoring. Nevertheless, the systematic analytical characterization presented here—including probit-based LoD estimation, inter-method agreement analysis, and identification of the viral load range in which discordance occurs—provides a transparent foundation for future optimization efforts. Establishing the performance boundaries of a laboratory-developed assay is itself a scientifically meaningful contribution, as it defines the conditions under which the assay can and cannot be reliably applied, and informs the design of improved next-generation assays targeting higher sensitivity thresholds.

It has been previously reported that measurement uncertainty increases at low copy numbers in real-time PCR-based tests and that evaluating analytical sensitivity using statistical modeling approaches allows for a more accurate definition of assay performance [19,20]. The LoD_95_ value determined by probit regression analysis in this study reflects the analytical detection capability of the developed assay near its detection limit. Such analytical characterization is particularly relevant for interpreting low-level viral load results in clinical testing. Minor deviations between individual experimental detection probabilities and the fitted probit curve are expected at very low template concentrations due to stochastic amplification effects and pipetting-related variation near the detection limit. However, clinical decision thresholds for initiating preemptive antiviral therapy in transplant recipients may vary depending on institutional protocols and assay characteristics. Therefore, the LoD value reported in this study should be interpreted within the context of analytical method comparison rather than as a predefined clinical decision threshold.

In this study, the inter-method relationship between the laboratory-developed CMV qPCR test and the Roche Cobas^®^ CMV test was evaluated based on log_10_-transformed quantitative results; the analyses were examined in terms of correlation, inter-method agreement, and the clinical acceptability of measurement differences. A weak and statisti-cally non-significant correlation was observed between the laboratory-developed CMV qPCR test and the reference method in Spearman rank correlation analysis performed on samples for which numerical quantitative results were available from both methods (ρ = 0.32; *p* = 0.22; *n* = 16). This finding may be related to the increased variability of quantitative measurements in the low viral load range and the resulting inter-method variation. The relatively lower positive percent agreement observed in this study may partly reflect differences in analytical sensitivity between the two assays, particularly at very low viral load levels. When assays differ in their limits of detection and calibration strategies, samples with viral loads close to the analytical detection threshold may yield discordant results due to stochastic amplification effects and methodological differences between platforms. Consistent with this interpretation, false-negative results were predominantly observed in samples with low-level viral loads near the assay’s limit of quantification. It is therefore important to contextualize the 62.2% PPA within this analytical framework: the majority of discordant results occurred in samples with viral loads below or near the LoD95 of the laboratory-developed assay (~1.6 × 10^4^ copies/mL), where detection is inherently probabilistic. This does not reflect a systematic failure of the assay in the clinically relevant high-viral-load range, but rather an expected consequence of the sensitivity gap between the two platforms. However, correlation analyses alone do not reflect inter-method absolute agreement; therefore, Bland–Altman agreement analysis was performed to assess the magnitude of differences between the measurements [18]. This limitation may reflect differences in analytical sensitivity between the assays, particularly in samples with viral loads close to the detection limit.

Bland–Altman agreement analysis was performed on a larger dataset (*n* = 21) by including paired detectable results after log_10_ transformation to assess the magnitude of differences between the two methods. The mean difference between the two methods was −0.48 log10 units, and the majority of measurements fell within the 95% limits of agreement.

Multicenter studies have shown that differences in reporting in IU/mL and copies/mL create difficulties in interpretation when comparing methods, and that inter-platform variations persist despite WHO standardization [21,22,23,24]. Due to the potential for the conversion between IU and copy number to be method-specific, depending on the target gene region, calibrator structure, and analytical platform, the use of a universal conversion coefficient is not recommended. Therefore, it is emphasized that in clinical practice, viral load results should be evaluated based on trends in change over time rather than a single absolute threshold value [11,25,26]. However, it should be acknowledged that in high-risk transplant recipients—particularly in CMV-seronegative recipients receiving organs or stem cells from CMV-seropositive donors (D+/R−)—any detectable reactivation event may carry significant risk of complications and require prompt clinical intervention. In such cases, early detection at low viremia levels is critical, and reliance on longitudinal trends alone may result in delayed treatment initiation. This further underscores the importance of high analytical sensitivity in assays intended for transplant monitoring. In this context, the analytical sensitivity of the assay (LoD95 ≈ 63.8 copies/µL, corresponding to approximately 1.6 × 10^4^ copies/mL) is above commonly used clinical decision thresholds reported in the literature. Therefore, the assay is unlikely to be suitable for early detection of CMV viremia in preemptive treatment strategies. Nevertheless, the assay may still provide reliable detection in cases with higher viral loads, suggesting potential utility as a rule-in tool for clinically significant CMV infection rather than early screening or precise quantitative monitoring. Although quantitative CMV PCR assays are widely used for viral load monitoring, their accessibility may be limited in certain laboratory settings due to cost and infrastructure requirements. Therefore, the present assay was developed as a cost-effective and accessible alternative rather than a replacement for highly standardized commercial platforms.

The primer-probe design used in this study targets the US17 region of the cytomegalovirus genome, which was selected based on evidence of sequence conservation across circulating CMV strains. In silico alignment analysis of available CMV genome sequences confirmed the absence of significant mismatches within the primer and probe binding regions, supporting the genetic—rather than merely functional—conservation of this target across clinical isolates (Supplementary Table S1). Studies evaluating the genetic content of the CMV genome have reported that genes in the US region display relatively limited sequence variability across clinical isolates compared to other genomic regions, which supports their utility as targets in molecular diagnostic applications [27]. Additionally, previous clinical studies have reported that real-time PCR approaches targeting the US17 region are applicable in clinical samples and can be used to monitor CMV viral load [28]. In contrast, it has been frequently reported in the literature that polymorphisms identified in the UL55 (glycoprotein B) or IE gene regions, which are often targeted, can negatively affect amplification efficiency by influencing primer-probe binding, especially in low viral load ranges [21,22]. Indeed, in Novak et al.’s study, it was shown that in the gB-targeted real-time PCR test, some samples showed negative or low levels of gB signal due to polymorphisms in the primer/probe region, which was inconsistent with the IE-targeted results [29]. Therefore, the selection of US17 as the target gene in this study reflects a conscious design preference based on its reported clinical applicability in the literature and its use as a target gene in real-time PCR applications across different sample types, rather than the absolute superiority of a specific gene region. This preference can be considered an approach that could contribute to more consistent and comparable results across different clinical materials by reducing the impact of amplification variability related to target gene polymorphisms.

Large cohort studies have shown that CMV disease poses a significant clinical and economic burden on transplant recipients [30]. This situation highlights that viral load monitoring plays a critical role not only in clinical management but also in reducing the burden on healthcare systems. These findings suggest that, rather than serving as a primary method in transplant centers that require early reactivation screening and precise viral load monitoring, the developed assay may have a more realistic role as a complementary tool in resource-limited laboratories, for rapid confirmation of patients presenting with high viral loads, or for detection of non-transplant, high-level CMV infections.

This study has some limitations. The single-center design of this study should be considered a limitation regarding the generalizability of the findings. Furthermore, the LoD95 of 63.8 copies/µL (~1.6 × 10^4^ copies/mL) is approximately one to two orders of magnitude higher than the detection thresholds of most commercial platforms. In transplant recipients, where early detection of reactivation is critical for initiating preemptive therapy, this sensitivity gap may result in missed low-level viremia events and delayed clinical intervention. Therefore, this assay should not be considered a replacement for high-sensitivity commercial platforms in transplant monitoring protocols. Additionally, analytical sensitivity experiments were performed using synthetic plasmid DNA (pUC57) diluted in water rather than CMV-negative human plasma matrix. While this approach is consistent with CLSI EP17-A2 guidelines for initial analytical characterization, it may not fully reflect real-world conditions, as plasma matrix effects and endogenous inhibitors could potentially increase background noise and raise the effective detection limit in clinical samples. Future studies should evaluate LoD using viral DNA diluted in CMV-negative plasma to better approximate clinical performance. Furthermore, detailed clinical metadata—including CMV serostatus at the time of transplantation, donor CMV serostatus, type of transplant (solid organ vs. hematopoietic stem cell), and prophylactic antiviral treatment status—were not systematically recorded for all patients included in this study. The absence of this information limits the ability to stratify assay performance across clinically relevant subgroups and should be addressed in future prospective studies. Moreover, while a cost-effectiveness rationale was provided for the development of this laboratory-developed assay, a formal cost comparison with commercial platforms was not performed in this study. Future studies should include a detailed economic analysis comparing reagent costs, infrastructure requirements, and per-sample expenditures between laboratory-developed and commercial assays, particularly in the context of resource-limited healthcare settings where transplantation programs are expanding but diagnostic infrastructure remains constrained. However, the detailed statistical modeling of analytical sensitivity and the simultaneous presentation of systematic comparisons with a reference method in clinical samples suggest that the results are reliable and complementary within the context of clinical monitoring. These findings should primarily be interpreted within the framework of analytical method comparison rather than as definitive evidence of clinical performance.

## 5. Conclusions

The laboratory-developed CMV qPCR assay evaluated in this study demonstrated measurable analytical performance; however, its sensitivity and agreement with the commercial reference method were limited, particularly at low viral load levels. For this assay to be considered suitable for routine clinical use in transplant monitoring, future optimization efforts should aim to achieve a LoD at least one to two orders of magnitude lower than the current LoD95 (~1.6 × 10^4^ copies/mL). Additionally, the current assay design is unlikely to be suitable for diagnosis of congenital CMV infection, where sensitivity requirements are even more stringent due to the typically low viral loads present in neonatal samples. Significant improvements in analytical sensitivity would be required before the assay could be applied in that clinical context. Future prospective and multicenter studies are needed to further define the role of laboratory-developed assays in clinical practice and to better understand the relationship between CMV viral load kinetics and clinical outcomes.

## Figures and Tables

**Table 1:** Evaluation of the analytical sensitivity (LoD95) of the CMV qPCR test using probit regression analysis.

Parameter	Estimate	Standard Error	z-Value	*p*-Value
Fixed (β_0_)	−5.35	0.57	−9.43	<0.001
Log_10_ (copy/µL) (β_1_)	3.88	0.38	10.20	<0.001

**Table 2:** Agreement analysis between the laboratory-developed CMV qPCR assay and the reference method (*n* = 100).

	Positive, *n*	Negative, *n*	Total
Laboratory-developed CMV qPCR test Positive	23 (TP)	5 (FP)	28
Laboratory-developed CMV qPCR test Negative	14 (FN)	58 (TN)	72
Total	37	63	100

TP True Positive, FP: False Positive, FN: False Negative, TN: True Negative, *n*: Number of samples.

**Table 3:** Bland-Altman agreement analysis results.

Parameter	Value
Number of matched samples (n)	21
Mean difference (log_10_)	−0.48
Standard deviation	0.93
Lower 95% limit of agreement (LoA)	−2.30
Upper 95% limit of agreement (LoA)	+1.35

## Data Availability

The raw data supporting the conclusions of this article will be made available by the corresponding author, Murat Aral, upon reasonable request.

## Supplementary Materials

The following supporting information can be downloaded at: https://www.mdpi.com/article/10.3390/microorganisms14051127/s1: Table S1. In silico compatibility analysis of US17 primer and probe binding regions. Table S2. MIQE 2.0 checklist for the CMV qPCR assay used in this study. Table S3. Diagnostic performance metrics of the laboratory-developed CMV qPCR test. Table S4. Agreement analysis between the laboratory-developed CMV qPCR test and the reference method using Cohen’s kappa. Table S5. CMV viral load results. Table S6. Samples included in quantitative correlation and Bland–Altman agreement analyses.

## Abbreviations

The following abbreviations are used in this manuscript:

CLSI	Clinical and Laboratory Standards Institute
CMV	Cytomegalovirus
DNA	Deoxyribonucleic acid
EDTA	Ethylenediaminetetraacetic acid
FAM	6-Carboxyfluorescein
FN	False negative
FP	False positive
IU	International units
LoA	Limits of agreement
LoD	Limit of detection
LoD95	Limit of detection at 95% probability
MIQE	Minimum Information for Publication of Quantitative Real-Time PCR Experiments
NPA	Negative percent agreement
NTC	No-template control
PCR	Polymerase chain reaction
PPA	Positive percent agreement
SPSS	Statistical Package for the Social Sciences
TN	True negative
TP	True positive
WHO	World Health Organization
qPCR	Quantitative real-time polymerase chain reaction
