# Supplementary material for: Analytical Performance and Inter-Method Agreement of a Laboratory-Developed CMV qPCR Assay in Clinical Plasma Samples
Source: Microorganisms. 2026 May 15;14(5):1127. doi: 10.3390/microorganisms14051127 (PMC13210100; doi:10.3390/microorganisms14051127)
Supplement: Supplementary file 1 [file microorganisms-14-01127-s001.zip › ST2.pdf]

Supplementary Table S2. MIQE 2.0 checklist for the CMV qPCR assay used in this study.

MIQE 2.0 Item	Description	Reported in Manuscript
Experimental design	Method comparison study evaluating analytical performance and inter-method agreement between a laboratory-developed CMV qPCR assay and a commercial reference assay	Methods section
Sample description	EDTA plasma samples obtained from patients with suspected or confirmed CMV infection	Methods – Study population
Sample size	100 clinical plasma samples	Methods – Study design
Nucleic acid extraction	Viral DNA extracted from plasma samples using standard extraction procedures prior to qPCR analysis	Methods – Sample preparation
Target gene	CMV US17 gene region	Methods – qPCR design
Primer sequences	Forward: TCTCTGTACCTCCCGCAAAA; Reverse: AGACAAACTCATCGCTTGGA	Methods – qPCR design
Probe sequence	FAM-TGACCTGGTTATCGTCACGCG-BHQ	Methods – qPCR design
Internal control	Human β-actin gene used to monitor extraction and amplification efficiency	Methods – qPCR design
qPCR chemistry	RapidXFire™ qPCR Master Mix (LGC Biosearch Technologies)	Methods
Reaction volume	20 μL total reaction volume	Methods
Primer concentration	500 nM for CMV primers	Methods
Probe concentration	125 nM for CMV probe	Methods
Thermal cycling conditions	Initial denaturation 95°C for 5 min, followed by 44 cycles of 95°C for 10 s and 60°C for 45 s	Methods
Fluorescence detection	Signal acquisition performed during the extension phase of each cycle	Methods
Analytical sensitivity	Limit of detection estimated using probit regression analysis	Methods / Results
LoD_{95}	Estimated at 63.8 copies/μL	Results
Dynamic range	Linear amplification observed between 10^2 and 10^6 copies/μL	Methods
Repeatability	Technical replicate variation low; day-to-day Ct differences ≤0.5 cycles	Methods
Statistical analysis	Spearman correlation, Bland–Altman agreement analysis, and diagnostic performance metrics (PPA, NPA, OPA) calculated	Statistical analysis section
Calibration materials	pUC57 plasmid containing CMV target sequence and Amplirun CMV DNA control used for calibration experiments	Methods
In silico analysis	Alignment analysis confirmed conservation of primer/probe binding regions within CMV genomes	Supplementary Table S1
