# Supplementary material for: Analytical Performance and Inter-Method Agreement of a Laboratory-Developed CMV qPCR Assay in Clinical Plasma Samples
Source: Microorganisms. 2026 May 15;14(5):1127. doi: 10.3390/microorganisms14051127 (PMC13210100; doi:10.3390/microorganisms14051127)
Supplement: Supplementary file 1 [file microorganisms-14-01127-s001.zip › ST6.pdf]

Supplementary Table S6. Samples included in quantitative correlation and Bland–Altman agreement analyses.

Sample ID	Included in Spearman correlation (n=16)	Included in Bland–Altman analysis (n=21)
8	✓	✓
14	✓	✓
15	✓	✓
17		✓
19		✓
26	✓	✓
27	✓	✓
30	✓	✓
31	✓	✓
32	✓	✓
40		✓
45	✓	✓
49	✓	✓
50	✓	✓
58		✓
61	✓	✓
66	✓	✓
69		✓
71	✓	✓
78	✓	✓
82	✓	✓
