# Supplementary material for: Analytical Performance and Inter-Method Agreement of a Laboratory-Developed CMV qPCR Assay in Clinical Plasma Samples
Source: Microorganisms. 2026 May 15;14(5):1127. doi: 10.3390/microorganisms14051127 (PMC13210100; doi:10.3390/microorganisms14051127)
Supplement: Supplementary file 1 [file microorganisms-14-01127-s001.zip › Supplementary Table S1.pdf]

Supplementary Table S1. In silico compatibility analysis of US17 primer and probe binding regions.

Region	Primer / Probe Sequence (5′–3′)	Number of CMV genomes analyzed (n)	Genomes with mismatches (n)	3′-end mismatches (n)	Explanation
Forward primer	TCTCTGTACCTCCCGCAAAA	346	2	0	3′-end evaluated
Probe	FAM-TGACCTGGTTATCGTCACGCG-BHQ	346	2	0	3′-end concept not applicable to probes
Reverse primer	AGACAAACTCATCGCTTGGA	346	2	0	3′-end evaluated

In silico PCR analyses were performed on 346 complete CMV genome sequences obtained from the NCBI Taxonomy database (TaxID: 10358). The designed primer–probe set was confirmed to target a highly conserved region. Complete sequence identity with the target region was observed in 99.4% of the analyzed genomes (n = 344). Exceptionally, amplification was not predicted in two genomes (Accession Nos: KU221096.1 and KU221098.1). Detailed sequence analysis demonstrated that this was not due to mismatches at primer binding sites but rather to structural genomic variations. These genomes correspond to genetically modified laboratory-derived strains (Human betaherpesvirus 5 Merlin strain variants RCMV1804 and RCMV1815), which contain large-scale deletions encompassing the US17 gene region. As a result, the absence of the target region in these genomes led to negative in silico amplification results. These findings confirm the high inclusivity of the designed assay for clinical and wild-type CMV strains.
