# Supplementary material for: Analytical Performance and Inter-Method Agreement of a Laboratory-Developed CMV qPCR Assay in Clinical Plasma Samples
Source: Microorganisms. 2026 May 15;14(5):1127. doi: 10.3390/microorganisms14051127 (PMC13210100; doi:10.3390/microorganisms14051127)
Supplement: Supplementary file 1 [file microorganisms-14-01127-s001.zip › Supplementary Table S3..pdf]

**Supplementary Table S3.** Diagnostic performance metrics of the laboratory-developed CMV qPCR test.

| Metric | Estimate (%) | 95% Confidence Interval |
|---|---|---|
| Positive Percent Agreement (PPA) | 62.2 | 46.1 – 75.9 |
| Negative Percent Agreement (NPA) | 92.1 | 82.7 – 96.6 |
| Overall Agreement (OA) | 81.0 | 72.2 – 87.5 |

Notes: PPA = TP / (TP + FN); NPA = TN / (TN + FP); OA = (TP + TN) / total samples. Confidence intervals were calculated using the Wilson score method.
