# Supplementary material for: Analytical Performance and Inter-Method Agreement of a Laboratory-Developed CMV qPCR Assay in Clinical Plasma Samples
Source: Microorganisms. 2026 May 15;14(5):1127. doi: 10.3390/microorganisms14051127 (PMC13210100; doi:10.3390/microorganisms14051127)
Supplement: Supplementary file 1 [file microorganisms-14-01127-s001.zip › Supplementary Table S4.pdf]

Supplementary Table S4. Agreement analysis between the laboratory-developed CMV qPCR test and the reference method using Cohen's kappa.

Parameter	Value
Cohen's kappa (κ)	0.57
95% Confidence Interval	0.39 – 0.73
Level of agreement	Moderate

Notes: Cohen's kappa was calculated based on the 2×2 contingency table (n = 100). The 95% confidence interval was estimated using bootstrap resampling.
