# Supplementary material for: Analytical Performance and Inter-Method Agreement of a Laboratory-Developed CMV qPCR Assay in Clinical Plasma Samples
Source: Microorganisms. 2026 May 15;14(5):1127. doi: 10.3390/microorganisms14051127 (PMC13210100; doi:10.3390/microorganisms14051127)
Supplement: Supplementary file 1 [file microorganisms-14-01127-s001.zip › Supplementary Table S5..pdf]

Supplementary Table S5. CMV viral load results.

Sample number	Reference CMV PCR Method (IU/mL)	Laboratory-developed CMV qPCR (Copies/mL)
1	Not detected	Not detected
2	Not detected	Not detected
3	82	Not detected
4	Not detected	Not detected
5	Not detected	Not detected
6	Not detected	Not detected
7	Not detected	Not detected
8	953	124
9	41	Not detected
10	Not detected	Not detected
11	Not detected	Not detected
12	Not detected	Not detected
13	Not detected	Not detected
14	1080	406
15	71	80
16	Not detected	Not detected
17	<34.5	37
18	Not detected	Not detected
19	<34.5	54
20	Not detected	Not detected
21	Not detected	21
22	Not detected	Not detected
23	Not detected	Not detected
24	Not detected	Not detected
25	Not detected	48
26	824	212
27	134	134
28	Not detected	18
29	Not detected	Not detected
30	62900	55100
31	39	54
32	223	438
33	Not detected	Not detected
34	Not detected	Not detected
35	Not detected	99
36	Not detected	Not detected

37	Not detected	Not detected
38	<34.5	Not detected
39	<34.5	Not detected
40	<34.5	3277
41	Not detected	Not detected
42	Not detected	Not detected
43	Not detected	24
44	Not detected	Not detected
45	865	210
46	Not detected	Not detected
47	Not detected	Not detected
48	Not detected	Not detected
49	57	531
50	73	51
51	<34.5	Not detected
52	Not detected	Not detected
53	Not detected	Not detected
54	Not detected	Not detected
55	Not detected	Not detected
56	Not detected	Not detected
57	Not detected	Not detected
58	<34.5	13
59	Not detected	Not detected
60	<34.5	Not detected
61	678	124
62	Not detected	Not detected
63	<34.5	Not detected
64	Not detected	Not detected
65	Not detected	Not detected
66	1880	1051
67	Not detected	Not detected
68	Not detected	Not detected
69	<34.5	10
70	<34.5	Not detected
71	22700	11710
72	Not detected	Not detected
73	<34.5	Not detected
74	Not detected	Not detected
75	Not detected	Not detected
76	Not detected	Not detected

77	Not detected	Not detected
78	1030	175
79	Not detected	Not detected
80	<34.5	Not detected
81	Not detected	Not detected
82	227	141
83	63	Not detected
84	Not detected	Not detected
85	Not detected	Not detected
86	<34.5	Not detected
87	5920	8
88	458	271
89	<34.5	Not detected
90	Not detected	Not detected
91	Not detected	Not detected
92	Not detected	Not detected
93	Not detected	Not detected
94	Not detected	Not detected
95	Not detected	Not detected
96	Not detected	Not detected
97	Not detected	Not detected
98	Not detected	Not detected
99	Not detected	Not detected
100	502	Not detected

Samples reported as <34.5 IU/mL by the reference Roche cobas® CMV assay were considered positive according to the manufacturer's instructions and were included in the positive category in the comparative analysis.
